# Combined Use of Wells Scores and D-dimer Levels for the Diagnosis of Deep Vein Thrombosis and Pulmonary Embolism in COVID-19: A Retrospective Cohort Study

**DOI:** 10.7759/cureus.17687

**Published:** 2021-09-03

**Authors:** Kavin Raj, Sanya Chandna, Sotirios G Doukas, Abi Watts, Keerthana Jyotheeswara Pillai, Anil Anandam, Dhruv Singh, Randy Nagarakanti, Kesavan Sankaramangalam

**Affiliations:** 1 Internal Medicine, Saint Peter’s University Hospital, New Brunswick, USA; 2 Endocrinology and Diabetes, Saint Peter’s University Hospital, New Brunswick, USA; 3 Electrophysiology, Robert Wood Johnson University Hospital, New Brunswick, USA; 4 Cardiology, West Virginia University, Morgantown, USA

**Keywords:** covid-19, deep vein thrombosis, pulmonary embolism, wells pe score, wells dvt score, d-dimers

## Abstract

Introduction

Deep vein thrombosis (DVT) and pulmonary embolism (PE) are key complications of coronavirus disease 2019 (COVID-19). The study's primary outcome was assessing the utility of Wells DVT, Wells PE scores, and D-dimers in diagnosing DVT and PE. Secondary outcomes were the risk factors for the development of PE and DVT in COVID-19 patients.

Materials and methods

We compared COVID-19 patients with a positive and negative lower extremity (LE) duplex. A similar approach was made for patients who underwent imaging for PE.

Results

The prevalence of PE was 23.8% (26 out of 109 patients), and the prevalence of DVT was 33% (35 out of 106). A D-dimer of 500 ng/mL had a sensitivity of 95.6% and 93.7% for the diagnosis of PE and DVT, respectively. A Wells DVT score of 3 points had a specificity of 92.9% and sensitivity of 8.8% for DVT diagnosis in COVID-19. A Wells PE score of 4 had a specificity of 100% and a sensitivity of 20% for the diagnosis of PE. The combined approach of using a Wells DVT score of 3 in suspected DVT and a Wells PE score of 4 in suspected PE and D-dimers of 500 ng/ml has a sensitivity of 94.2% and 96.1%, respectively.

In the suspected DVT group, male gender (OR 3.88, 95% CI 1.55-9.7, P=0.004), lower body mass index (BMI) (OR 0.92, 95% CI 0.86-0.99, P=0.037), antiplatelet use (OR 0.19, 95% CI 0.04-0.88, P=0.035), systolic blood pressure *≤*100 mmhg (OR 4.96, 95% CI 1.37-17.86, P=0.014), absolute lymphocytes *≤*1 (OR 2.57, 95% CI 1.07-6.12, P=0.033), D-dimer *≥*500 ng/ml (OR 6.42, 95% CI 1.40-29.38, P=0.016), blood urea nitrogen (BUN) *≥*20 mg/dl (OR 2.33, 95% CI 1.00-5.41, P=0.048), and intubation (OR 3.32, 95% CI 1.26-8.78, P=0.015) were found to be statistically significant for DVT.

In the suspected PE group, history of cancer (OR 10.69, 95% CI 1.06-107.74, P=0.044), total WBC count (OR 1.07, 95% CI 0.95-1.21, P=0.032), platelets *≥* 400,000 (OR 5.13, 95% CI 1.79-14.68, P=0.002), D-dimer levels *≥* 500 ng/ml (OR 25.47, 95% CI 3.27-197.97, P=0.002), Wells PE score (OR 2.46, 95% CI 1.50-4.06, P<0.001), pulmonary embolism rule-out criteria (PERC) score (OR 1.79, 95% CI 1.05-3.05, P=0.054), and Sequential Organ Failure Assessment (SOFA) score (OR 1.91, 95% CI 1.16-3.12, P=0.002) were statistically significant.

Conclusions

The combined approach of using a Wells DVT score of 3 in suspected DVT and Wells PE score of 4 in suspected PE and D-dimers of 500 ng/ml may be used to diagnose PE and DVT in COVID-19. Venous thromboembolism (VTE) occurrence in COVID-19 is associated with non-traditional risk factors such as intubation and higher severity of systemic inflammation, and these patients may benefit from more aggressive testing for VTE.

## Introduction

Coronavirus disease 2019 (COVID-19) is a pandemic caused by severe acute respiratory syndrome coronavirus 2 (SARS-CoV-2). There is a broad spectrum of disease manifestations, including asymptomatic shedding, acute upper respiratory tract infection, multilobar pneumonia, acute respiratory distress syndrome, and death [1]. However, COVID-19 is also characterized by thrombotic complications such as pulmonary embolism (PE) and deep vein thrombosis (DVT) [2]. The pathogenesis of COVID-19-associated venous thromboembolism (VTE) involves hypercoagulability and endothelial damage, as shown by different studies [3-8]. Several studies have shown that the cumulative incidence of VTE in COVID-19 patients varies from 3.1% to as much as 40% [2,9-21].

VTE is screened based on a combination of Wells scores, D-dimers, and Clinical gestalt [22-25]. This approach enables clinicians to screen patients effectively and avoid unnecessary testing, as shown by previous studies [22-25]. The utility of this algorithm has not been robustly tested in the COVID-19 setting [26-28]. Therefore, we designed this study to validate the combined approach of using D-dimers and Wells scores to diagnose VTE in COVID-19 patients.

## Materials and methods

Data collection

The charts of 1300 COVID-19 patients admitted from March 1, 2020, to December 1, 2020, were reviewed retrospectively. Patients who had imaging studies for DVT or PE within 90 days of COVID-19 illness were included. The patients with lower extremity (LE) duplex were included in the suspected DVT group, and patients with CT pulmonary angiogram (CT-PA) or V/Q scan were included in the suspected PE group. COVID-19 disease is diagnosed with active symptoms of COVID-19 and positive SARS-CoV-2 RT-PCR by nasopharyngeal swab.

Variables include demographics, risk factors for VTE such as ongoing malignancy, tobacco smoking, estrogen use, active pregnancy, recent surgery (within 12 weeks), previous history of DVT, history, physical exam, vitals, laboratory investigations, and outcomes. All the clinical variables, including Wells scores, were calculated based on the information in the charts on the day of imaging for VTE, and D-dimers were obtained within seven days prior to the day of imaging for VTE. There was high suspicion for VTE in COVID-19 patients in our institution, and hence clinicians obtained imaging for VTE based on clinical judgment even when D-dimer or Wells scores were low. Wells scores were calculated based on the information in the charts by three trained investigators.

The study's primary outcome was assessing the utility of Wells DVT, Wells PE scores, and D-dimers in diagnosing DVT and PE. Secondary outcomes were the risk factors for the development of PE and DVT in COVID-19 patients. We also included two serial D-dimers values within 10 days before VTE imaging to study the role D-dimer trend in diagnosing VTE.

Statistical analysis

A two-sample t-test was used to analyze normally distributed data, and the Wilcoxon rank-sum test was used to analyze non-normally distributed continuous data. Logistic regression was used to analyze categorical variables and to generate odds ratios for continuous variables. The multivariate analysis was done using penalized logistic regression as proposed by Firth. The final model was chosen based on the Akakai information criterion. Receiver operating curves, sensitivity, and specificity were calculated for D-dimer levels, Wells PE, and Wells DVT scores. Odds ratios (ORs) and corresponding 95% confidence intervals (CIs) were presented as effective measures. Any variable with missing data of more than 10% was not included in the analysis. A p-value of less than or equal to 0.05 was considered significant. All data were analyzed with Stata/SE version 17 (StataCorp. 2021. Stata Statistical Software: Release 17. College Station, TX: StataCorp LLC.).

## Results

Prevalence of VTE

Out of the 1300 patients, 210 met the study criteria. Thus, 106 subjects were selected for the suspected DVT group, and 109 patients were selected for the suspected PE group. Seven patients were positive for both PE and DVT. These patients were included as cases in both the groups but not as a control in either group. In the suspected PE group, the prevalence of PE in the patients who underwent CT-PA or V/Q scan was 23.8% (26 out of 109 patients). On the other hand, the prevalence of DVT in patients who underwent duplex was 33% (35 out of 106).

Clinical features and risk factors of VTE

In the suspected DVT group, DVT was usually diagnosed within a few days after hospitalization (Median 4, IQR 1-10) and in the second week of COVID-19 illness (Median 11, IQR 4-22). In the suspected PE group, PE was usually diagnosed on the first day of presentation (Median 1, IQR 1-5) and in the second week of COVID-19 illness (Median 9, IQR 4-22). Neither the clinical features nor the classic risk factors for VTE were found to have a statistically significant association in both the DVT and PE subgroups. All the patients in our cohort received prophylactic anticoagulation on the day of admission. We also reported the subset of patients who were on any dose of prophylactic heparin or enoxaparin for at least five days before VTE testing (Tables 1-2).

**Table 1:** Key results on univariable analysis in suspected DVT group † Mean standard deviation for normally distributed data; ‡ Median (Interquartile range) for non-normally distributed data Calf symptoms of DVT include unilateral or bilateral calf swelling, redness, or calf pain and prophylactic anticoagulation was mentioned only if patients were on any dose of prophylactic heparin or enoxaparin for at least five days prior to VTE testing. These criteria were chosen as almost all patients receive prophylactic anticoagulation unless patients have significant current bleeding. As most patients were tested for VTE during the initial days of hospitalization, they were not included as being prophylactically anticoagulated. µ Labs were within one to seven days of being tested for VTE with most values being drawn one to three days prior to being tested for VTE. DVT: deep vein thrombosis; SOFA: Sequential Organ Failure Assessment

Variables	Duplex positive N=35	Duplex negative N=71	Odds ratio (95% CI)	P-value
Age, years	63±14 †	61±18	1.00(0.98-1.03)	0.588
Male sex, n (%)	27(77%)	33(46.4%)	3.88(1.55-9.71)	0.004
Body mass index, kg/m^2^	27.9±4.1	30.6±6.9	0.92(0.86-0.99)	0.037
Calf symptoms of DVT	6(17.1%)	7(9.8%)	1.89(0.58-6.12)	0.288
Bedbound	5(14.2%)	10(14%)	1.01(0.31-3.24)	0.978
Active solid cancer	0	2(2.8%)		
Active hematologic cancer	0	2(2.8%)		
History of cancer	1(2.8%)	4(5.6%)	0.49(0.05-4.58)	0.534
Past history of VTE	1(2.8%)	4(5.6%)	0.49(0.05-4.58)	0.534
Full dose anticoagulation	2(6.2%)	5(7.3%)	0.84(0.15-4.58)	0.840
Prophylactic anticoagulation >5 days ^&^	10(29.4%)	25(35.2%)	0.76(0.31-1.85)	0.556
Antiplatelet use	2(5.7%)	17(23.9%)	0.19(0.04-0.88)	0.035
Systolic blood pressure, mmHg	115(100-125)	125(110-140)	0.96(0.94-0.98)	0.005
Absolute lymphocytes, 10^9^/L	0.7(0.4-1.2)	1(0.6-1.4)	0.55(0.27-1.12)	0.040
D-dimer, ng/ml	2937(1471-3680)	721(396-1363)	1.00(1.00-1.00)	P<0.001
Blood urea nitrogen, mg/dl	33(15-62)	19(12-34)	1.02(1.00-1.04)	0.018
Wells DVT Score	0(1-2)	0(1-2)	1.36(0.91-2.05)	0.150
Wells DVT score=0	11(32.3%)	29(40.8%)		
Wells DVT score=1	6(17.6%)	20(28.1%)		
Wells DVT score=2	14(41.1%)	17(23.9%)		
Wells DVT score=3	3(8.8%)	5(7%)		
SOFA, n (%)	2(1-2)	1(1-2)	1.11(0.90-1.36)	0.051
SOFA score=0	1(2.9%)	8(11.2%)		
SOFA score=1	15(44.1%)	40(56.3%)		
SOFA score=2	10(29.4%)	10(14%)		
SOFA score>2	8(23.5%)	13(18.3%)		
ICU, n (%)	15(44%)	21(29.5%)	1.87(0.80-4.38)	0.144
Death, n (%)	11(32.3%)	21(29.5%)	1.13(0.47-2.74)	0.773
Intubation, n (%)	12(35.2%)	10(14%)	3.32(1.26-8.78)	0.015

**Table 2:** Key results on univariable analysis for PE occurrence † Median (interquartile range) for non-normally distributed data ‡ Mean standard deviation for normally distributed data ! Calf symptoms of DVT include unilateral or bilateral calf swelling, redness, or calf pain & Prophylactic anticoagulation is if patients were on any dose of prophylactic heparin or enoxaparin for at least five days prior to VTE testing. This criterion was chosen as almost all patients receive prophylactic anticoagulation unless patients have significant current bleeding. As most patients were tested for VTE during the initial days of hospitalization, they were not included as being prophylactically anticoagulated. "Vitals include average vitals on the day of the imaging for VTE µ Labs were within 1 to 7 days of being tested for VTE with most values being drawn 1 to 3 days prior to being tested for VTE DVT: deep vein thrombosis; SOFA: Sequential Organ Failure Assessment; VTE: venous thromboembolism; PE: pulmonary embolism; PERC: pulmonary embolism rule-out criteria; CT-PA: CT pulmonary angiogram

Variables	Case (CT-PA or V/Q positive) N=26	Control (CT-PA negative) N=83	OR (95% CI)	P-value
Age, years	63 (47-78) †	55 (44-70)	1.01 (0.99-1.04)	0.167
Calf symptoms of DVT!	2 (7.6%)	5 (5.88%)	1.64 (0.28-9.54)	0.579
History of cancer	3 (11.5%)	1 (1.1%)	10.69 (1.06-107.74)	0.044
Past history of VTE	1 (3.8%)	3 (3.5%)	1.06 (0.10-10.71)	0.956
Full dose anticoagulation	2 (7.6%)	7 (8.4%)	0.88 (0.17-4.53)	0.879
Prophylactic anticoagulation ^&^	6 (23%)	24 (28.2%)	0.73 (0.26-2.06)	0.562
Antiplatelet use	3 (11.5%)	10 (11.7%)	0.95 (0.24-3.75)	0.944
Heart rate ^"^	92±15	90±16	1.00 (0.97-1.03)	0.714
Oxygen saturation, %	96±2	95±2	1.10 (0.89-1.35)	0.370
Temperature, F	98.5 (98.2-99.5)	99 (98.5-100)	0.74 (0.49-1.11)	0.028
Rales	1 (3.8%)	19 (22.3%)	0.13 (0.01-1.06)	0.057
Loud P2	1 (3.8%)	0		
Total count, 10^9^/L ^µ^^¶^	8.55 (7.2-11.3)	7.3 (5.7-8.9)	1.07 (0.95-1.21)	0.032
Platelet, 10^9^/L	348 (203-414)	255 (182-339)	1.00 (1.00-1.00)	0.027
Absolute lymphocytes, 10^9^/L	1.22 (1-1.7)	1.06 (0.7-1.44)	1.11 (0.79-1.57)	0.028
D-dimer, ng/ml	2247 (1711-3680)	468 (267-865)	1.00 (1.00-1.00)	<0.001
Wells PE Score	2 (1.5-3)	1 (1-1.5)	2.46 (1.50-4.06)	<0.001
<2	9 (34.6%)	70(84.3%)		
2-6	15 (57.6%)	7 (8.4%)		
>6	2 (7.6%)	0		
PERC, n (%)	1 (1-3)	1 (1-1.5)	1.79 (1.05-3.05)	0.054
0	2 (7.6%)	9 (10.8%)		
1	12 (46.1%)	53 (63.8%)		
>1	12 (46.1%)	21 (25.3%)		
SOFA, n (%)	1 (1-2)	1 (1-2)	1.91 (1.16-3.12)	0.002
0	1 (3.8%)	19 (22.8%)		
1	12 (46.1%)	47 (56.6%)		
2	8 (30.7%)	31 (37.3%)		
>2	5 (19.2%)	4 (4.8%)		
ICU, n (%)	3 (11.5%)	8 (9.4%)	1.48 (0.35-6.20)	0.592
Death, n (%)	2 (7.6%)	5 (5.8%)	1.71 (0.29-9.97)	0.547
Intubation, n (%)	1 (3.8%)	1 (1.1%)	3.41 (0.20-56.68)	0.391

Possible risk factors for DVT events

In the suspected DVT group, male gender (OR 3.88, 95% CI 1.55-9.7, P=0.004), lower BMI (OR 0.92, 95% CI 0.86-0.99, P=0.037), antiplatelet use (OR 0.19, 95% CI 0.04-0.88, P=0.035), systolic blood pressure 100 mmHg (OR 4.96, 95% CI 1.37-17.86, P=0.014), absolute lymphocytes 1 (OR 2.57, 95% CI 1.07-6.12, P=0.033), D-dimer 500 ng/ml (OR 6.42, 95% CI 1.40-29.38, P=0.016), BUN 20 mg/dl (OR 2.33, 95% CI 1.00-5.41, P=0.048), intubation (OR 3.32, 95% CI 1.26-8.78, P=0.015) were found to be statistically significant for DVT (Table 1). Wells DVT score was not significant (OR 1.36, 95% CI 0.91-2.05, P=0.150). On multivariate analysis using penalized logistic regression, the D-dimer levels (OR 1.00, 95% CI 1.00-1.00, P=0.003) and systolic blood pressure (OR 0.99, 95% CI 0.93-0.99, P=0.024) remained significant.

Possible risk factors for PE events

In the suspected PE group, history of cancer (OR 10.69, 95% CI 1.06-107.74, P=0.044), temperature 98.4F (OR 2.87, 95% CI 1.10-7.21, P=0.030), absence of rales (OR 0.13, 95% CI 0.01-1.06, P=0.057), total WBC count (OR 1.07, 95% CI 0.95-1.21, P=0.032), platelets 400,000 (OR 5.13, 95% CI 1.79-14.68, P=0.002), D-dimer levels 500 ng/ml (OR 25.47, 95% CI 3.27-197.97, P=0.002), Wells PE score (OR 2.46, 95% CI 1.50-4.06, P<0.001), PERC score (OR 1.79, 95% CI 1.05-3.05, P=0.054), and SOFA score (OR 1.91, 95% CI 1.16-3.12, P=0.002) were statistically significant (Table 2). On multivariate analysis using penalised logistic regression D-dimer levels (OR 1.00, 95% CI 1.00-1.00, P<0.001) and Wells PE score (OR 2.44, 95% CI 1.12-5.33, P=0.024) remained statistically significant.

Accuracy of D-dimers in VTE diagnosis

**Figure 1:**
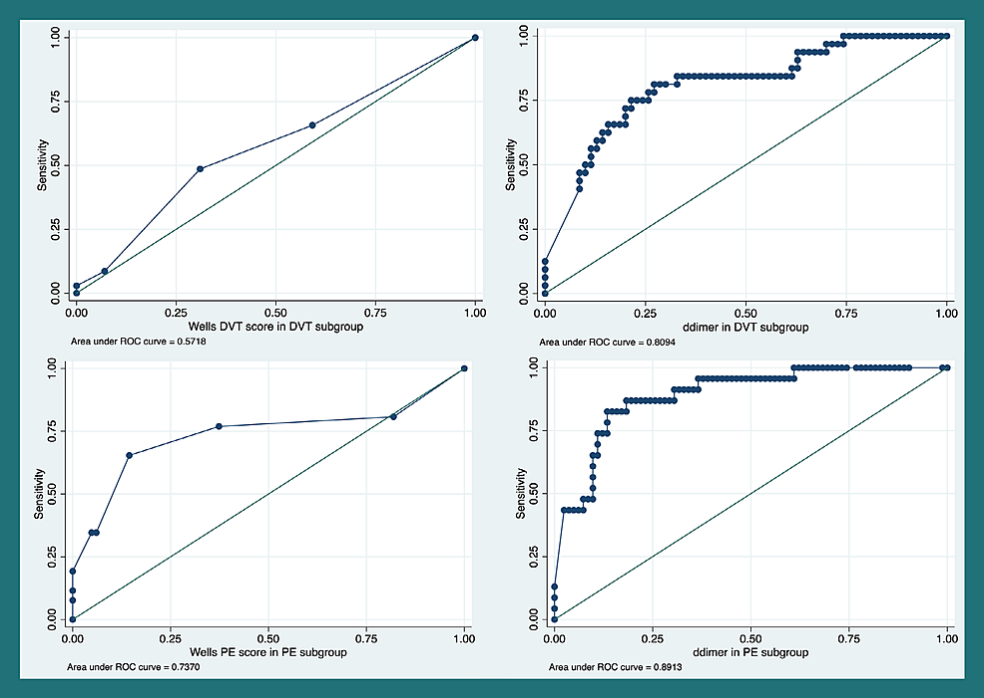
ROC analysis for Wells scores and D-dimers ROC: receiver operating characteristic

In the suspected PE group, receiver operating characteristic (ROC) analysis for D-Dimer and Wells PE score showed an area under the curve (AUC) of 0.89 and 0.73, respectively (Figure 1). A D-dimer cut-off of 1500 ng/ml had a sensitivity of 82.6% and a specificity of 85.4% for PE diagnosis. Assuming a disease prevalence of 10% (20-22), the negative predictive value for a D-dimer of 1500 ng/ml is 97.8%, and the positive predictive value is 38.5%. For a D-dimer of 500 ng/ml, the sensitivity is 95.6%, specificity is 53.6%, the negative predictive value is 99.1%, and the positive predictive value is 18.6%. In the suspected DVT group, ROC analysis for DVT using D-dimer levels and Wells DVT score showed an area under the curve (AUC) of 0.80 and 0.58, respectively. A D-dimer of 500 ng/ml has a sensitivity of 93.7 and specificity of 30% for DVT. Assuming a prevalence of 10%, the negative predictive value is 97.7% and the positive predictive value is 12.9% (Tables 3-4).

**Table 3:** Accuracy of Wells scores and D-dimer for prediction of PE PE: pulmonary embolism

	Sensitivity (%)	Specificity (%)	PPV (%)	NPV(%)
D-dimer of 500 ng/ml	95.6	53.6	18.6	99.1
D-dimer of 1500 ng/ml	82.6	85.4	38.5	97.8
Wells PE score of 4	20	100	100	91.8
Wells PE score of 6	8	100	100	90.7
Serial D-dimers	94.1	32.5	13.4	98

**Table 4:** Accuracy of Wells scores and D-dimer for the prediction of DVT DVT: deep vein thrombosis; PPV: positive predictive value; NPV: negative predictive value

	Sensitivity (%)	Specificity (%)	PPV (%)	NPV (%)
D-dimer of 500 ng/ml	93.7	30	12.9	97.7
D-dimer of 1500 ng/ml	75	77.1	26.7	96.5
Wells DVT score of 2	50	69	15.2	92.5
Wells DVT score of 3	8.8	92.9	12.2	90.1
Serial D-dimers	94.1	32.5	13.4	98

Accuracy of Wells scores for DVT events

**Figure 2:**
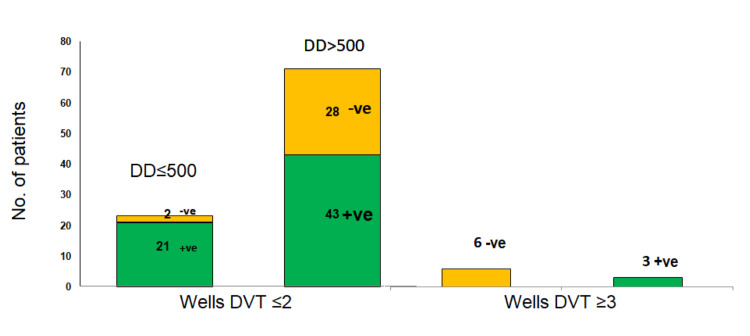
Distribution of D-dimer and Wells DVT scores in COVID-19 DVT +ve represents DVT positive. -ve represents DVT negative.

**Figure 3:**
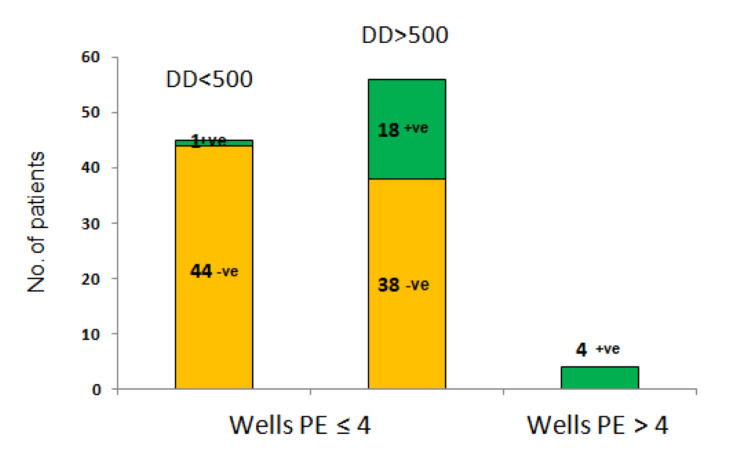
Distribution of Wells PE scores and D-dimers in COVID-19 PE +ve represents DVT positive. -ve represents DVT negative. PE: pulmonary embolism; DVT: deep vein thrombosis

In the suspected DVT group, 6.6% of subjects had Wells DVT scores of 3 or above. In this group, the DVT prevalence was 28.5%. In the remaining patients with a Wells DVT score of 2 or less, the DVT prevalence was 8.6% in D-dimer 500 ng/ml and 43% in D-dimer 500 ng/ml. Notably, two patients with a D-dimer level of <500 ng/ml and a Wells DVT score of less than 2 had a positive LE duplex for DVT (Table 3, Figures 2-3). Overall the combined sensitivity of D-dimer of 500 ng/ml and Wells DVT score of 3 was 94.2%. A Wells DVT score of 2 has a sensitivity of 50% and specificity of 69% for DVT diagnosis. A Wells DVT score of 3 has a sensitivity of 8.8% and a specificity of 92.9% for DVT diagnosis.

Accuracy of Wells scores for PE events

In the suspected PE group, only 4.5% of patients in the suspected PE group had a Wells PE score of 4 or above. However, in this group, the PE prevalence was 100%. In patients who had a Wells score of 4 or less, PE prevalence was 2.2% for D-dimer <500 ng/ml, 6.8% for D-Dimer in between 500-1500 ng/ml, and 60% when D-dimer was >1500 ng/ml, respectively. A Wells PE score of 4 has a sensitivity of 20% and specificity of 100% for PE diagnosis. A Wells PE score of 6 has a sensitivity of 8% and a specificity of 100% for PE diagnosis (Table 3).

Serial D-dimers for VTE diagnosis

Within 10 days before diagnostic imaging for VTE, serial D-dimer was available for 96 patients: 17 cases (DVT and PE positive) and 79 controls. Of the 17 cases, five were positive for PE, and 13 were positive for DVT. Up-trending D-dimer levels had a sensitivity of 94.1% and a specificity of 32.5%. Assuming a disease prevalence of 10%, the positive predictive value is 13.4%, and the negative predictive value is 98% (Table 3)

## Discussion

Prevalence of VTE in COVID-19

Early literature suggested a 20% to 40% incidence of DVT and PE in COVID-19 [3,10-1]. However, several more extensive studies showed an incidence of 3 to 8% [12-23]. In our study, prevalence is 23.8% for PE and 33% for DVT, and the overall prevalence of VTE is 28.3% (61/215). However, our prevalence doesn't represent the true prevalence of VTE occurrence in COVID-19 as not all the 1300 patients had imaging for VTE.

Diagnostic challenges in COVID-19-associated DVT

Before the COVID-19 pandemic, Wells DVT scores and D-dimer levels were routinely used to determine the pre-test probability for DVT. The modified Wells DVT score includes clinical features and risk factors of DVT [24]. In patients with Wells DVT score 2, high-sensitivity D-dimer testing is recommended. If D-dimer is less than 500 ng/ml, DVT is considered to be ruled out, and no further testing is recommended. If the D-dimer level is more than 500 ng/ml, LE duplex should be done. Patients with a Wells DVT score of 3 should undergo a duplex without a D-Dimer test [22-25].

Wells DVT score was not significant in our study. So far, no studies have directly explored the utility of Wells DVT scores in the COVID-19 population. Most of the patients in our study did not have the typical symptomatology and risk factors present in the existent Wells DVT scoring. We believe that extensive prospective studies exploring other parameters in COVID-19 patients, such as intubation, SOFA scores, and severity of COVID-19, could potentially lead to a modified Wells DVT score that could be used for COVID-19 patients. A D-dimer level cut-off of 500 ng/ml has a good sensitivity of 93.7% and a specificity of 30%. Our findings align with the Wells DVT score algorithm, suggesting that patients with a Wells DVT score of 2 or less should get D-dimer levels, and if D-dimer levels are more than 500 ng/ml, duplex LE should be done.

In addition, similar to other studies, male sex, intubation, and higher BUN and SOFA scores are associated with DVT in COVID-19 [12,15,17,20]. These parameters reflect the severity of the disease, and hence clinicians should consider aggressive testing for DVT in this patient with severe COVID-19 disease.

Diagnostic challenges in COVID-19-associated PE

None of the classic clinical features or risk factors for PE were associated with PE occurrence in the PE group, likely because PE in COVID-19 was mostly clinically silent. In general, in patients with suspected PE, a modified Wells score, which classifies patients into low risk (Wells score<2), intermediate-risk (Wells score 2-6), and high risk (Wells score>6) categories, should be done. In patients with low-risk scores, the use of PERC is applied. If the PERC score is zero, no further testing is needed. If the PERC score is one or above, then D-dimer testing is recommended. D-dimer testing is also recommended in patients with intermediate wells PE scores. A D-dimer of more than 500 ng/ml is considered high risk for PE, and CT-PA or V/Q scan is recommended. Patients with high modified Wells scores should be assessed directly with CT-PA or V/Q scan [23-25].

In our subjects, only 11 out of 109 patients had zero scores on PERC. This was because two of the PERC variables, such as oxygen saturation less than 95% and tachycardia, were highly prevalent in our COVID-19 patients. Also, two patients who had zero scores on PERC were found to have PE. Therefore, the utility of PERC scores might be limited in COVID-19.

In our subjects, a D-dimer of 1500 had a high specificity. Hence, we strongly recommend testing for PE when the D-dimer value is above 1500. However, the existing D-dimer cut of 500 ng/ml has a higher sensitivity and should be used to screen for PE when Wells scores are less than 4. Overall, the combined approach of using Wells PE and D-dimer level of 500 ng/ml successfully predicted PE in all but one of our patients (25 out of 26) with a sensitivity of 96%.

Studies are conflicting about the usefulness of Wells PE scores and D-dimers in predicting PE in COVID-19 [26-28]. Our study supports the combined approach of using Wells PE score and D-dimer in COVID-19 patients. However, prospective and large-scale studies are needed to establish the usefulness in COVID-19 patients definitively.

Role of serial D-dimers

Although only half of our patients had serial D-dimers, our findings suggest that serial D-dimer levels may be predictive for VTE. If they are increasing, Imaging for VTE can be performed. On the other hand, if D-dimers decrease, duplex LE may not be necessary. However, clinicians should be aware of the low specificity of D-dimers and the different causes for elevations in D-dimers, such as systemic inflammatory response syndrome (SIRS) response, sepsis, and renal failure, which are common in COVID-19 patients.

Study limitations

The main limitation of our study is its retrospective nature. Wells scores are calculated based on information in the charts, which may have lead to measurement bias. Prospective studies in predicting VTE in the COVID-19 setting can be very challenging during a pandemic. Hence, the authors accept this as the first step to studying the utility of these scores in the COVID-19 setting. The prevalence in our study is not true prevalence, as patients were screened based on clinical suspicion, similar to most prior studies. Some patients received empiric anticoagulation over the suspicion of PE but were not included in this study, as they did not have diagnostic testing. The burden of missing variables is highest for serial D-dimers, as they were done in only half of the patients and should be interpreted cautiously.

## Conclusions

A high wells DVT or PE score may be suggestive of PE or DVT, but such high scores occur less frequently in COVID-19-associated VTE as these VTEs are clinically silent and are not associated with the classic risk factors. Conversely, the clinical features of COVID-19, such as chest pain, hypoxia, and tachycardia, overlap with PE. This leads to erroneously giving 3 points on Wells PE scores. Thus, in our study, Wells PE scores appear to have more value than Wells DVT scores in COVID-19 patients.

Low D-dimer levels might be helpful to rule out VTE, and we recommend clinicians continue to use a D-dimer cut-off of 500 ng/dl in screening for DVT and PE. However, high D-dimers are more commonly seen in COVID-19 patients regardless of VTE. Serial D-dimers have the same issue, but they may be superior to a single D-dimer value. Serial D-dimers need to be evaluated in large prospective studies. Until more extensive studies are available, we recommend that clinicians continue to use the combined approach of Wells score and D-dimers but be aware of its limitations in the COVID-19 setting.
